# Chatbot-assisted therapy for patients with methamphetamine use disorder: a preliminary randomized controlled trial

**DOI:** 10.3389/fpsyt.2023.1159399

**Published:** 2023-07-07

**Authors:** Lee Chun-Hung, Liaw Guan-Hsiung, Yang Wu-Chuan, Liu Yu-Hsin

**Affiliations:** ^1^Department of Information Engineering, I-Shou University, Kaohsiung, Taiwan; ^2^Jianan Psychiatric Center, Ministry of Health and Welfare (MOHW), Tainan, Taiwan; ^3^King's College London, Florence Nightingale School of Nursing & Midwifery, London, United Kingdom

**Keywords:** mHealth, chatbot, methamphetamine, addiction, substance abuse

## Abstract

**Background:**

Methamphetamine (MA) use disorder is associated with a large public health burden. Despite the therapeutic effects of psychosocial interventions based on current evidence, finding an approach to retain patients in treatment remains a real-world challenge. The rapid development of mobile health (mHealth) systems suggests the potential to provide real-time personalized care at any time and from any location, minimize barriers to treatment, maximize use, and promote the dissemination of accessible therapeutic tools in at-risk populations. Our study aimed to investigate the feasibility and effectiveness of chatbots for the treatment of MA use disorder.

**Method:**

The inclusion criteria were (a) a diagnosis of MA use disorder as defined by the DSM-5, (b) age between 18 and 65 years, (c) no acute exacerbation of severe mental illness during the initial assessment, such as schizophrenia or bipolar I disorder, (d) willingness to participate in standard outpatient treatment for ≥ 6 months, and (e) an Android phone. Participants were randomly allocated to either a chatbot-assisted therapy via smartphone (CAT) group or a control group following simple randomization procedures (computerized random numbers) without blinding. All participants were followed up for 6 months. Treatment retention and monthly urine test results were analyzed as outcome measures. Participants' satisfaction with CAT was also assessed.

**Results:**

In total, 50 and 49 participants were allocated to the CAT and control groups, respectively. There were no significant differences in retention time between the two treatment groups (*df* = 1, *p* = 0.099). The CAT group had fewer MA-positive urine samples than the control group (19.5% vs. 29.6%, *F* = 9.116, *p* = 0.003). The proportion of MA-positive urine samples was positively correlated with the frequency of MA use (*r* = 0.323, *p* = 0.001), severity of MA use disorder (*r* = 0.364, *p* < 0.001), and polysubstance use (*r* = 0.212, *p* = 0.035), and negatively correlated with readiness to change (*r* = −0.330, *p* = 0.001). Totally 55 participants completed the study at the 6-month follow-up and 60% reported relative satisfaction.

**Conclusion:**

Participants in this study had favorable acceptance and generally positive outcomes, which indicates that chatbot is feasible for treating people who use MA.

## 1. Introduction

Methamphetamine (MA), a psychostimulant with high abuse potential, can result in physical and psychological harm (1, 2). Acute MA intoxication often leads to agitation and violence (3), and chronic use may cause infection (4), heart failure (5), and mental health problems such as depression (6), psychosis (7), and suicidality (8). Furthermore, MA use places a significant burden on social services and criminal justice systems due to legal offenses and other hazardous behaviors (9). The pervasive methamphetamine use problem presents a multifaceted global public health issue. According to the United Nations Office on Drugs and Crime (UNODC) World Drug Report 2022, approximately 34 million people, or 0.43% of the global population, are estimated as past-year users of amphetamines (which includes methamphetamine) in 2020 (10). These figures are likely an underestimate given the hidden nature of substance use. Moreover, drug misuse rose to 30% in the past decade and COVID-19 could worsen the situation (11). Methamphetamine use is particularly concentrated in several regions. In North America, the United States has reported a stark increase in methamphetamine-related fatalities and hospital admissions. Southeast Asia and the Pacific regions, including countries such as Taiwan, the Philippines, and Australia, also report high methamphetamine usage. Simultaneously, Eastern and Southern Africa are witnessing a growing trend in methamphetamine trafficking and consumption. Given this expansive and complex landscape, providing adequate treatment for patients with MA use disorder is crucial.

Current evidence supports the efficacy of face-to-face psychosocial interventions, such as the Matrix Model program (12), cognitive behavioral therapy (13), relapse prevention (14), and contingency management (15, 16), as first-line treatment due to the lack of effective pharmacotherapies (17). However, MA-associated psychosis may contribute to difficulties in delivering services (18–20). Moreover, patients with mental health comorbidities, polysubstance use, unemployment, or housing problems are less likely to engage in treatment (21). Thus, the accessibility of resources for MA use disorder treatment remains an issue.

Completing treatment and undergoing more intensive interventions are important for reducing MA use and improving psychiatric symptoms (22). Nevertheless, treatment attendance and completion rates are variable and decline as the intensity of treatment increases (23–25). Dropping out of addiction treatment is common due to drug cravings, psychological distress, lack of motivation to change, psychiatric comorbidities, the severity of the substance use disorder, and maladaptive personality functioning (26–30). Thus, engaging patients with MA use disorder and providing immediate support to retain patients remains a challenge. In response, researchers and therapists have focused on developing and evaluating programs that can reduce barriers to treatment initiation, improve patient compliance, increase treatment adherence and retention, and reduce patient dropout rates.

Smartphones have become ubiquitous, and most individuals own and use them daily. As smartphones have evolved, they have become a platform for mental health support through mobile health (mHealth) systems in the form of various applications and services that can assist individuals in managing their mental wellbeing. Delivering interventions through mHealth offers several benefits, including continuous therapeutic support regardless of time and location, low barriers to access due to geographic isolation, and convenient dissemination to at-risk populations (31, 32). This approach not only provides personalized care instantly but also represents a cost-effective solution to enhance patients' self-efficacy and support recovery in mental healthcare (33–35). Additionally, mHealth-based treatment has been shown to result in a similar therapeutic alliance and treatment satisfaction compared with face-to-face interventions (36). Moreover, researchers have demonstrated the effectiveness of mHealth in enrolling patients into treatment programs, providing psychosocial interventions during treatment, reducing risky behaviors, and promoting adherence to substance use disorder treatment (33, 37). A systematic review of 11 randomized controlled trials (2 for opiate, 4 for alcohol, 3 for MA, and 2 for polysubstance abuse) assessed the acceptability, feasibility, and effectiveness of text messaging interventions for individuals with drug and alcohol dependence (38). The review found that improved clinical outcomes, medication adherence, and engagement with peer support groups were observed in most studies with technology-based interventions, providing a promising strategy to confront barriers such as stigma, privacy concerns, and being alone without assistance (39).

Chatbots are computer programs that simulate human conversation and can be accessed via smartphones, laptops, and tablets. In the field of mHealth, chatbots can assist with various tasks, including mental health and addiction management (40, 41). Advances in technology have led to the development of chatbot-delivered psychotherapy, a form of mental health treatment that uses AI to provide support and guidance to individuals in need. The use of chatbots in psychotherapy offers several benefits, such as increased accessibility, convenience, and affordability (41, 42). A 2018 randomized controlled trial found that a chatbot-delivered CBT program was effective in reducing symptoms of post-traumatic stress disorder and alcohol use in military veterans (43). Lucas et al. also found that a chatbot was effective in eliciting sensitive information and demonstrating higher symptom disclosure, as well as decreasing fear of self-disclosure (44). Additionally, a meta-analysis of 11 trials by Lim et al. found that chatbot-delivered psychotherapy was effective in reducing depressive symptoms (45). Another study of chatbot-delivered interventions for gambling disorder revealed that it significantly decreased severity (*p* = 0.01 and 0.003), cravings (*p* = 0.03 and.02), frequency (*p* = 0.01 and.004), and expenditure (*p* = 0.04 and 0.003) of gambling problems at postintervention and follow-up (46). Moreover, a randomized controlled trial of a therapeutic chatbot (Woebot-SUD) during the COVID-19 pandemic showed that it significantly reduced the number of occasions of substance use (*p* = 0.039) (47). Overall, these findings suggest that chatbot-delivered psychotherapy can effectively reduce symptoms of various mental health conditions, including anxiety, depression, post-traumatic stress disorder, gambling disorder, and substance use disorders.

For patients with MA use disorder, effective treatment using mHealth systems is still being developed, and most treatments utilize text-messaging systems. A pilot study by Reback et al. showed significant improvement in substance use among 52 men who have sex with men after a 2-week text messaging intervention (48). The results showed that at follow-up, participants had significant improvements in MA use, such as a decrease in the frequency of use (*p* < 0.01), an increase in abstinence (13.3% vs. 48.9%, *p* < 0.001), and an increase in MA-free periods (*p* < 0.01). Additionally, Kioleian et al. developed a text messaging intervention as an adjunct to cognitive behavioral group therapy for MA users and demonstrated its feasibility and potential (49). A randomized controlled trial by Moore et al. showed that text messaging intervention could improve antiretroviral therapy adherence (OR = 8.31, *p* < 0.01) and reduce MA use (intervention: 14.4 days vs. active control: 22.0 days, *p* = 0.05) among individuals with MA use disorder (50). Takano et al. conducted a small study comparing the effects of online relapse prevention courses with outpatient treatment and found that individuals who participated for longer than 1 year were likely familiar with MA treatment concepts and interventions (51). Thus, previous studies have mainly focused on text messaging as a method of intervention; however, with the advancements in artificial intelligence (AI) such as chatbots, research on more current forms of digital mental health is lacking. Therefore, the feasibility and effectiveness of chatbot-delivered treatment for patients with MA use disorder should be investigated.

Compared with traditional text messaging interventions, chatbots offer a more human-like experience and can be programmed to provide personalized support based on an individual's specific needs (52). For example, a chatbot can provide different responses according to the individual's stage of recovery. Chatbots also have the advantages of 24/7 availability, anonymity, and relatively low cost, making them an affordable option for treatment providers. This digital approach can reach users in remote or stigmatized communities, provide continual support, and deliver personalized guidance, enhancing the efficiency and scope of existing interventions. With the COVID-19 pandemic, face-to-face therapy sessions have become harder to access, and chatbots have become a crucial means of providing mental health support. The integration of chatbot-assisted therapy into broader drug policy could play a crucial role in mitigating the global methamphetamine problem by offering accessible, stigma-free, and immediate support to users worldwide. However, few studies have focused on the utilization of chatbots among patients with substance use disorders in clinical settings. The present study tested the hypothesis that participants receiving chatbot-assisted treatment (CAT), which delivers treatment reminders, interactive mindfulness-based relapse prevention (MBRP), and recovery skills training, as adjuvant treatment, would have similar or better outcomes compared with patients receiving standard care. This study proposes a chatbot-assisted treatment strategy, an innovative and adaptable intervention that leverages AI to provide personalized, accessible, and stigma-free support overcoming geographic disparity, thus potentially augmenting the effectiveness of traditional therapeutic approaches. This approach's unique features are particularly relevant for addressing the uneven distribution and diverse nature of global drug use, offering a promising direction for future drug policy and public health initiatives. The study outcomes included treatment retention and negative urine drug test results. We also performed a systematic evaluation of adherence, engagement, and subject satisfaction with CAT.

## 2. Methods

### 2.1. Study design and setting

We performed a randomized, placebo-controlled, parallel-group study without blinding (1:1 experimental to control group ratio), according to the CONSORT Statement 2010. The experimental group received CAT, and both groups received standard treatment provided by the facility. No interim analyses for efficacy or futility were performed. This study was registered at https://doi.org/10.1186/ISRCTN16586487, and the study was approved by the Institutional Review Board of Jianan Psychiatric Center (IRB-18-017). The study population was recruited from outpatients of the Addiction Unit at Jianan Psychiatric Center from January 2018 to July 2019.

### 2.2. Recruitment and participants

**Figure 1:**
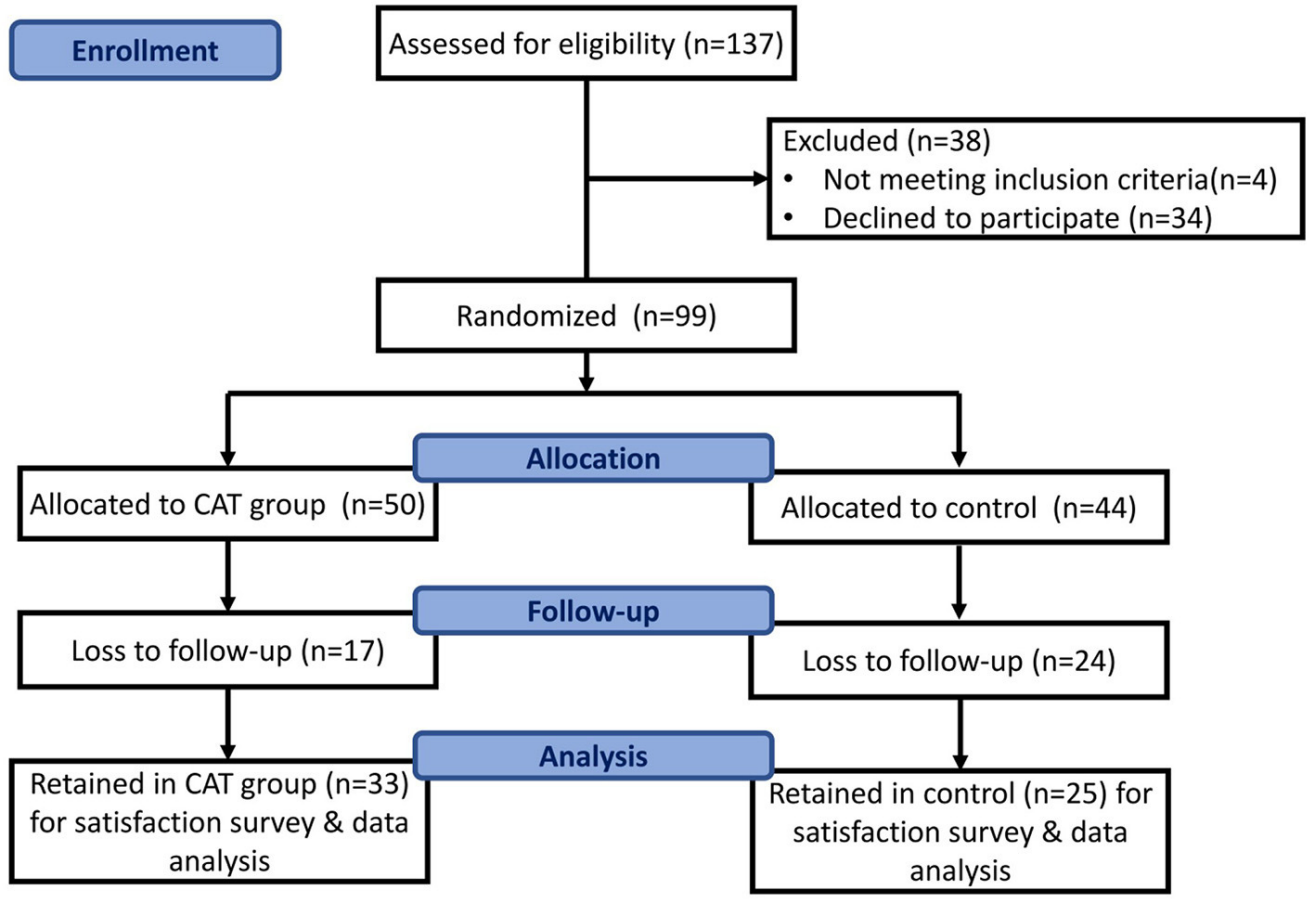
Flowchart of the study inclusion process.

The inclusion criteria for this study were as follows: (a) a diagnosis of MA use disorder as defined by the DSM-5, (b) age 18–65 years, (c) no acute exacerbation of severe physical or mental illnesses, such as schizophrenia or bipolar I disorder during the initial assessment, (d) a willingness to participate in standard outpatient treatment for a minimum of 6 months, and (e) possession of an Android phone for the app assessment. Participants were provided an explanation of the study and an opportunity to provide informed consent. Those who were unwilling to participate were incarcerated during the study period or hospitalized due to physical or mental illnesses and were considered to have dropped out of the study. The CAT was introduced as an adjuvant treatment for MA use disorder and promoted through flyers, email, and via our case managers to attract users. Users could download the Line app and add our chatbot on it. After informed consent was obtained, the participants who agreed to enroll were given an information sheet and asked to provide their contact details. Case managers then scheduled further visits for the study. The patient screening and recruitment process is shown in Figure 1. All participants underwent comprehensive interviews conducted by trained psychiatrists to gather their demographic data (such as name, age, sex, marital status, education level, and employment status) and drug use history (including age at first use, duration of use, number of unique events in a criminal record, and current dose in the previous month). Diagnostic interviews were also performed to confirm a diagnosis of substance use disorder. The severity of MA use was defined by the number of DSM-5 criteria met (2–3 criteria: mild, 4–5 criteria: moderate, ≥6 criteria: severe).

### 2.3. Randomization and outcome measurements

Eligible patients were randomly assigned via computerized randomization to one of the two groups (1:1 ratio) on the day of enrollment after baseline measurements were taken. The randomization was prepared by an investigator who was not involved in the trial, and the allocation sequence was concealed from the researcher until after the participants completed their baseline assessments. The allocation was revealed by a contact independent of the recruitment process when they opened the corresponding number concealed in an envelope.

Participants attended outpatient visits at the time of randomization and monthly intervals. Treatment retention and results from monthly urine tests were used as primary outcome measurements. The readiness to change was assessed using the University of Rhode Island Change Assessment (URICA) scale before randomization and the end of the study (53). The participants' satisfaction and app quality were also assessed as secondary outcomes. The Mobile Phone Use Questionnaire consists of four items, including “satisfied with receiving CAT”, “receiving CAT helped me to deal with addiction problems”, “felt someone cared by receiving CAT”, and “would suggest that other patients receive CAT”, which were used to reveal the patients' experiences and satisfaction using CAT for MA use disorder treatment. The quality of the chatbot app was evaluated by three reviewers (one psychiatrist, one mental health nurse, and one information engineer) using the Mobile Application Rating Scale (MARS) (54). All participants were required to visit Jianan Psychiatric Center every month, at which time, urine samples were collected. We utilized an immunoassay-based urine drug test specifically designed to detect methamphetamine use. Participants submitted urine samples every month throughout the intervention. Urine tests were conducted monthly for 1 year, yielding at least a total of 12 tests per participant. Physical and mental status examinations were performed as necessary. The study followed the participants for 6 months.

### 2.4. Psychological theory and intervention design

MBRP is an evidence-based treatment approach that combines mindfulness practices with traditional relapse prevention strategies to help individuals who struggle with substance abuse, including MA abuse (55–59). MBRP can increase acceptance, compassion, and resilience toward cravings and help maintain recovery from addiction.

Studies have investigated the effectiveness of MBRP for MA abuse and found that it may be an effective treatment approach. For example, an 8-week MBRP program was found to improve mindfulness, self-compassion, and psychological wellbeing, and reduce MA use in individuals who completed the program (59). Another randomized controlled trial showed that those who received MBRP in addition to standard substance abuse treatment had greater reductions in MA use and cravings compared with those who received standard treatment alone (58). The emphasis on mindfulness practices can help individuals develop greater control over their urges to use the drug, manage cravings and negative emotions more effectively, and lead to reductions in drug use after treatment. Between-session practices may contribute to initial increases in mindfulness and improved clinical outcomes after treatment (56, 60). In our study, we adapted MBRP as a part of the treatment module and used a chatbot with an online MBRP course to assist participants in engaging in between-session practice, rather than standard care.

**Figure 2:**
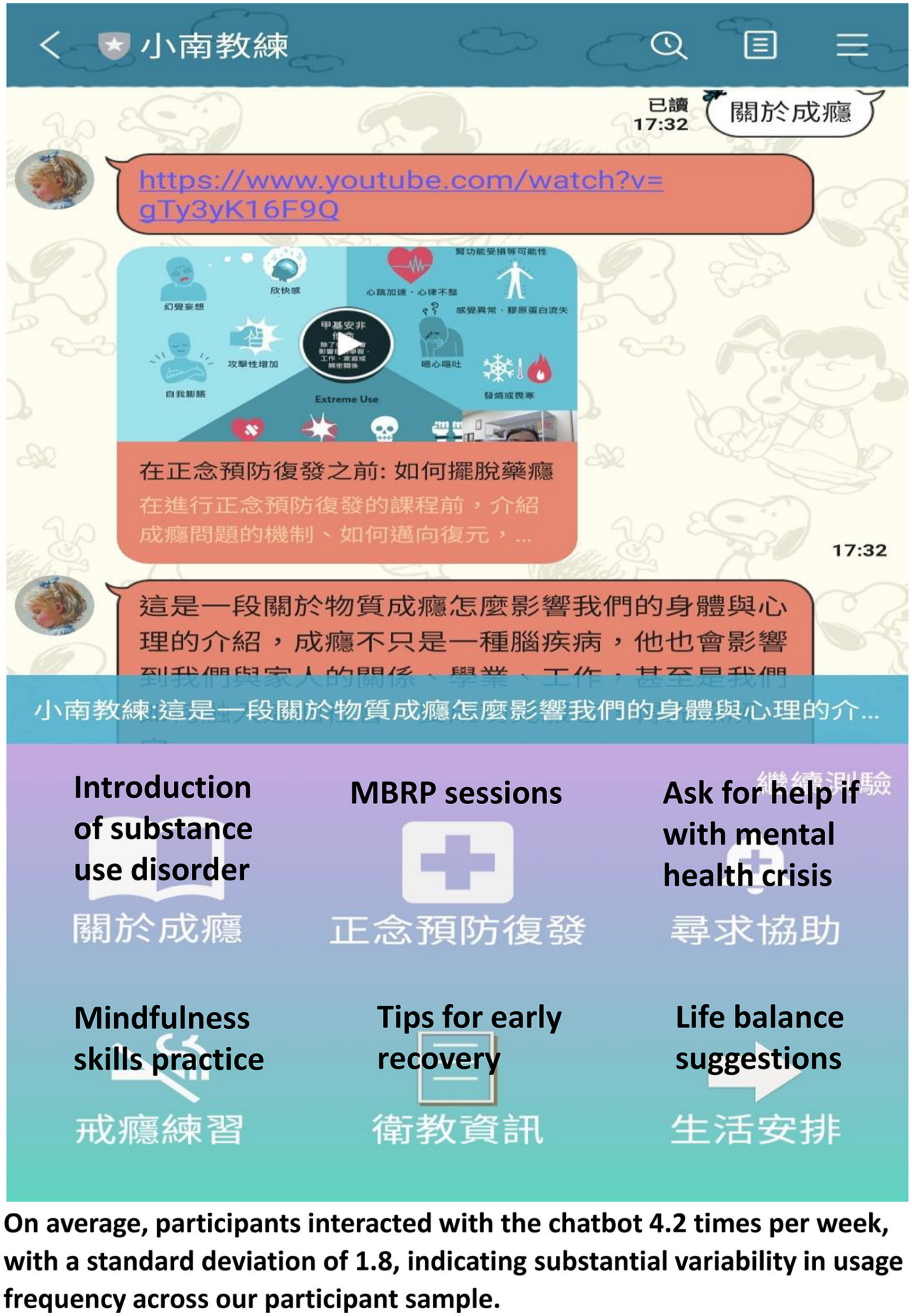
Example of the CAT program. CAT, chatbot-assisted treatment.

The chatbot application was meticulously developed by our research team following a collaborative and iterative design process. The initial stage of development involved identifying and defining the specific intervention problem that the chatbot was to address. This process was undertaken by a diverse focus group composed of mental healthcare professionals (consisting of a psychiatrist and a mental health nurse) and two technical experts. Utilizing the valuable insights gathered from these discussions, our development team proceeded to create a prototype of the chatbot. The design integrated crucial features such as natural language processing and machine learning capabilities, which were harmoniously interfaced with the Line chatbot platform. Following the development phase, the chatbot was deployed on the Line platform, which was deemed a suitable medium for access by our focus group and anticipated user population. During this preliminary deployment stage, both members of the focus group and case managers from Jianan Psychiatric Centers were invited to interact with the chatbot in a realistic setting, thus closely mimicking the user experience of the eventual participants. Throughout this process, data pertaining to user interaction patterns and general feedback were systematically gathered and meticulously analyzed. Following this preliminary testing phase, the gathered insights were presented to the focus group for further discussion and feedback. Based on this invaluable feedback, the chatbot underwent necessary adjustments and refinements, ensuring optimal functionality and user interaction. Subsequently, the refined chatbot was officially deployed for use within the study. The chatbot used in our study was tailored to the audience's level of familiarity with the subject matter and was based on artificial intelligence in the communication software, LINE. Figure 2 shows a dashboard outlining the different paths, including MBRP sessions, lifestyle concerns, and reminders for conversations with the chatbot natural language processing and deep learning were applied in the dialog system (61). Figure 2 illustrates the chatbot's key roles in facilitating various aspects of treatment, enhancing user understanding and engagement throughout the recovery process. The first function of the chatbot, as depicted on the left, is providing an introduction to substance use disorder. This includes the definition, common symptoms, and potential causes and consequences, aiming to improve the user's awareness and understanding of their condition. The subsequent section details the chatbot's role in delivering mindfulness-based relapse prevention strategies. It shows how the chatbot guides users in learning and applying these techniques, assisting them in mitigating cravings and managing triggers. Next, this figure illustrates how the chatbot facilitates mindfulness skills practice. Users are guided through various mindfulness exercises and are encouraged to integrate these practices into their daily lives. Following this, the chatbot offers tips for early recovery, providing users with practical advice and strategies to navigate the initial challenges of abstaining from substance use. The penultimate section of Figure 2 demonstrates that participants can reach out to helpful resources in times of mental health crises through the crisis helpline established by our government. Finally, it concludes with the chatbot's function of suggesting strategies for life balance. This includes advice on maintaining a healthy lifestyle, managing stress, and promoting overall wellbeing, enhancing users' ability to maintain recovery and improve their quality of life.

The MBRP sessions consisted of eight components: (1) automatic pilot and relapse, (2) awareness of triggers and cravings, (3) mindfulness in daily life, (4) mindfulness in high-risk situations, (5) acceptance and skillful action, (6) seeing thoughts as thoughts, (7) self-care and lifestyle balance, and (8) social support. Participants in the experimental group were introduced to the app and provided with a suggested schedule for 2 months of weekly MBRP sessions, along with home practices. They were instructed to access video recordings of eight MBRP sessions featuring various mindfulness practices through the chatbot and received weekly treatment reminders that coincided with their schedule.

Individuals in the experimental group were reminded of their MBRP sessions with the message, “We have an MBRP session to attend this week. It will help your recovery”, and were encouraged to continue with positive feedback, such as “Keep going” and “Good job”, after completing a session. The chatbot also assessed the participants' mood daily using the-5 Brief Symptom Rating Scale-5 (BSRS-5) questionnaire (62) and provided feedback, as well as guided meditations. The participants in the experimental group could access the materials anytime throughout the 6-month study period. An electronic reporting system allowed the researchers to view summaries of their patients' app activity. Participants who attended more than 80% of the online treatment sessions were considered to have completed treatment. In addition to MBRP, the chatbot provided psycho-educative materials, such as early recovery skills adapted from the Matrix Model manual (63) and information on maintaining a balanced life without drug use, aiming to build basic knowledge of addiction problems and help prevent early relapse. The participants in the control group received standard MBRP, which was conducted in person over eight sessions with the same themes as those in the experimental group. During these sessions, Master's level therapists summarized the progress in each group and provided reminder handouts. The therapists were also supervised to ensure adherence to the protocol. In addition, the psycho-educative materials provided to the experimental group were also provided to those in the control group after each session. After the study was complete, participants in the control group were invited to use the chatbot.

### 2.5. Statistical analysis

The sample size calculation for the study was conducted using G^*^Power software. The computations were based on a two-sided hypothesis test with a significance level of 0.005 and a power of 80% to detect an odds ratio of 1.0 for the outcome variables. The estimated proportion of 60% versus 30% in each group was based on previous studies investigating the effectiveness of psychosocial interventions for individuals with MA use disorder (30, 64, 65). The results indicated that a sample size of 42 participants per group was necessary.

All data analyses were performed using SPSS 21.0. Descriptive statistics, including the mean and standard deviation for quantitative variables, and the frequency and percentage for categorical variables, were calculated for all sociodemographic, clinical, and psychosocial variables. Differences in characteristics between the study groups were examined using a one-way analysis of variance (ANOVA) and a chi-squared test. The data were analyzed using an intention-to-treat approach, and missing data were assumed to be missing completely at random. The criterion for significance was set at *p* < 0.05.

The differences in the objectively measured adherence values of clinical data gathered at follow-up were examined using a one-way ANOVA. A matched paired *t*-test was performed to compare the response rates of the two groups regarding psychotherapy appointment attendance and drug urine test results. Pearson's correlation test was applied to evaluate significant trends. The cumulative retention in treatment was calculated using the Kaplan–Meier method with a log-rank test, based on the number of days in treatment from initiation until the patient quit or the end of the 6-month follow-up period. Variables that were significantly associated with retention were included in the multivariate Cox regression analysis and presented as odds ratios with 95% confidence intervals. Logistic and linear regression analyses were used to analyze the differences between potential predictor variables (such as age, sex, education, employment, and severity according to DSM-5 criteria) and the proportion of negative urine samples. Subgroup analyses according to addiction severity or polysubstance use were performed using Cox hazards models, and a *p* < 0.05 was considered statistically significant.

## 3. Results

### 3.1. Participants' demographic characteristics

Of the 99 participants, 81 were men (81.8%) and 18 were women (18.2%). All participants underwent a survey, treatment intervention, and follow-up observation. Moreover, 16 participants in the experimental group and 18 participants in the control group were assigned to our OPD (outpatient department) for prosecution and addiction treatment, and no significant distribution was observed between the groups. Additionally, 17 participants from the experimental group and 24 from the control group dropped out, resulting in data from 41 participants being available for the intention-to-treat analysis. The reasons for dropout included incarceration (experimental group *n* = 6, control group *n* = 11), hospitalization (control group *n* = 2), and lack of contact (experimental group *n* = 11, control group *n* = 11). No deviations from the protocol were noted, and all data were synthesized for further analysis. The mean age was 37.00 ± 10.40 years (range: 20–69 years), and most patients were between 31 and 40 years of age (Table 1). The mean number of years of education was 11.02 ± 2.43 years (range: 6–16 years), and most participants were male (81.8%, 81/99), had a high school degree (57.6%, 57/99), were single (53.5%, 53/99), and were employed (80.8%, 80/99). There were 18 participants diagnosed with mental illness and revealed no difference between the two groups (experimental group *n* = 8, control group=10, *p* = 0.611). As for comorbidities among our participants, the most common psychiatric diagnoses were major depressive disorder (*n* = 12), alcohol use disorder (*n* = 10), and anxiety disorder (*n* = 3). Six participants were diagnosed with at least two comorbid mental disorders. No significant differences were observed between the two groups in terms of categorical or continuous variables (*p*>0.05). The overall retention rate was 58.6% (58/99).

**Table 1:** Descriptive statistics of study variables: demographic data.

**Category**	**Experimental group (*n* = 50)**	**Control group (*n* = 49)**	***P* value**
Gender	M: 39, F: 11	M: 42, F: 7	0.320
Age (years old)	38.12 ± 9.17	35.98 ± 11.51	0.308
**Education (years)**			
Lower (0–9)	16	15	0.126
Secondary (10–12)	28	29	
Higher (≥13)	6	5	
**Marital status**			0.401
Single	26	27	
Married	13	10	
Divorced	9	12	
Others	2	0	
**Employment**			0.602
Job	39	41	
Jobless	10	8	
**Monthly income (NT/month)**			0.689
>45,000	10	11	
34,000–45,000	2	3	
24,000–34,000	10	10	
<24,000	27	25	

### 3.2. Drug use history

The participants' drug use history is summarized in Table 2. The average duration of MA use was 4.27 ± 6.14 years (range: 0.1–30 years). At baseline, the average daily amount of MA consumed per week was 0.72 ± 0.46 g (range: 0.04–5.62 g). Among all participants, 27 (27.6%) reported using MA more than four times per week, 16 (16.2%) had a history of polysubstance use, and 18 (18.2%) had comorbid severe mental illnesses, such as schizophrenia, bipolar disorder, and major depressive disorder. Moreover, 45.5% (45/99) of participants exhibited mild, 23.2% (23/99) exhibited moderate, and 31.4% (31/99) exhibited severe MA use disorder. The stage-of-change statistics were as follows: 14.1% (14/99) in precontemplation, 35.4% (35/99) in contemplation, 45.5% (45/99) in preparation, and 5.1% (5/99) in action. No significant differences were observed regarding drug use history between the two groups in terms of categorical or continuous variables (*p* > 0.05). However, a significant positive correlation was found between the participants' age and duration of MA use (*r* = 0.248, *p* = 0.013). Additionally, a significant positive correlation was found between the severity of MA use and the duration of MA use (*r* = 0.206, *p* = 0.041) and weekly expenditure (*r* = 0.272, *p* = 0.015).

**Table 2:** Descriptive statistics of study variables: drug use history.

**Category**	**Experimental group (*n* = 50)**	**Control group (*n* = 49)**	***P* value**
Duration of MA use (years)	4.15 ± 5.50	4.39 ± 6.79	0.295
Weekly expenses of MA use	2,128.21 ± 4,012.26 NT	1290.00 ± 1,304.44 NT	0.213
**Frequency**			
Frequent users	13	14	0.126
Infrequent users	36	35	
Polysubstance use	7 (14.29%)	9 (18.00%)	0.616
**Severity (DSM-5 criterions)**			0.602
Mild (2-3 criterions)	20	25	0.541
Moderate (4–5 criterions)	13	10	
Severe (≧6 criterions)	17	14	
**Stage of change**			0.278
Precomtemplation	5	9	
Preparation	15	20	
Determination	27	18	
Action	3	2	

### 3.3. Outcome variables

**Figure 3:**
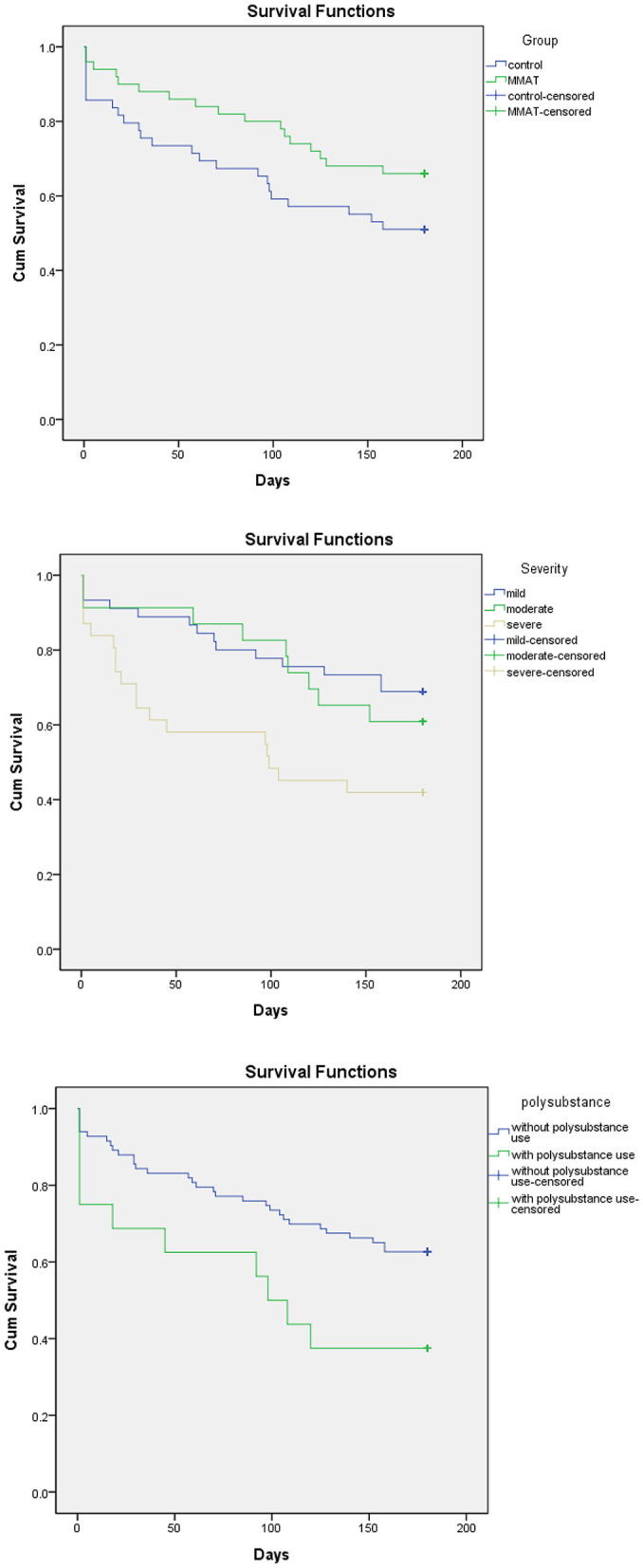
Variables related to treatment retention. CAT, chatbot-assisted treatment; MA, methamphetamine.

The average retention time was 142.42 ± 60.54 days in the experimental group and 118.12 ± 73.41 days in the control group. The 6-month completion rates for the experimental and control groups were 66% and 51%, respectively. Figure 3 illustrates that no significant difference was observed in treatment retention between the CAT and control groups (*df* = 1, *p* = 0.099). However, participants with more severe MA use disorder (*df* = 2, *p* = 0.023), low readiness to change (*df* = 3*, p* < 0.001), and polysubstance use (*df* = 1, *p* = 0.030) tended to have shorter treatment retention periods (Figure 3). The multivariate Cox regression analyses of other categorical predictors for retention revealed no significant differences. Since we excluded those who demonstrated acute exacerbation of mental illness that could potentially impede their ability to participate fully in the study, psychiatric comorbidities did not affect the retention (*df* = 1, *p* = 0.12).

A total of 378 urine samples were collected: 209 in the CAT group and 169 in the control group. The experimental group had fewer MA-positive urine samples than the control group (19.5% vs. 29.6%, *F* = 9.116, *p* = 0.003). The proportion of MA-positive urine samples was positively correlated with the frequency of MA use (*r* = 0.323, *p* = 0.001), severity of MA use disorder (*r* = 0.364, *p* < 0.001), and polysubstance use (*r* = 0.212, *p* = 0.035), and was negatively correlated with readiness to change (*r* = 0.330, *p* = 0.001). When the pretest scores were used as covariates, an ANOVA revealed that the intervention group only had higher posttest scores in the contemplation subscale of the URICA (*F* = 5.6, *p* = 0.012). No significant differences in scores for readiness to change and the URICA subscale were observed between the experimental and control groups. The average attendance rate for MBRP was 75.1% in the experimental group and 60.8% in the control group. No significant differences in treatment attendance, completion, or retention were observed between patients referred for prosecution treatment and those who were not. No harm or unintended effects were reported in either group.

### 3.4. Feasibility, participant satisfaction, and app quality

**Figure 4:**
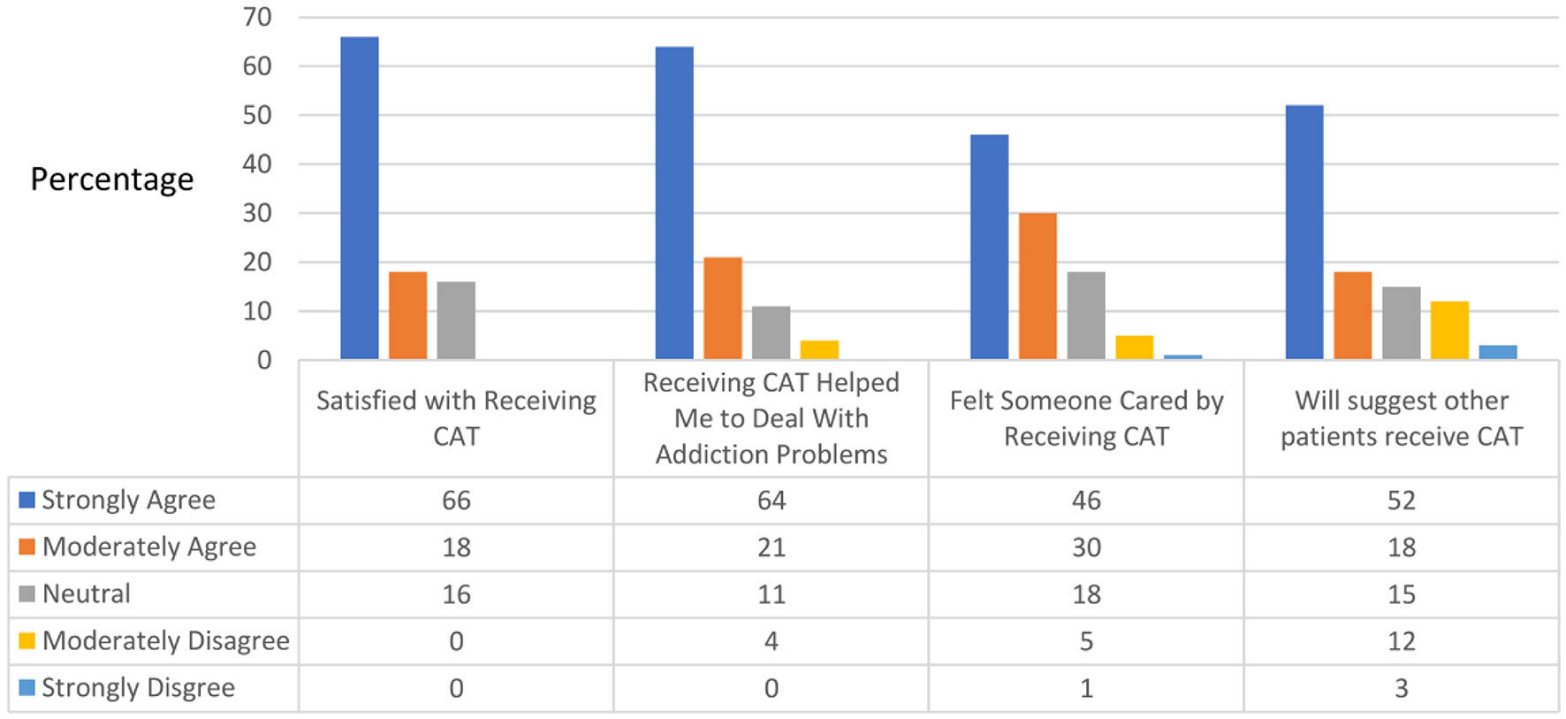
Satisfaction with CAT. CAT, chatbot-assisted treatment.

Of the 33 participants who completed the study treatment program, 29 completed the Mobile Phone Use Questionnaire at the end of the 6-month follow-up. The experimental group reported relative satisfaction with the chatbot, as indicated by strong-to-moderate agreement with the following statements: “Satisfied with receiving CAT” (84%), “Receiving CAT helped me to deal with addiction problems” (85%), “Felt someone cared by receiving CAT” (76%), and “Would suggest that other patients receive CAT” (67%) (Figure 4). Only two patients reported technical difficulties in receiving CAT, while the rest reported strong or moderate agreement that the chatbot was easy to use. Upon review by three experts, the mean app quality score evaluated by the MARS was 4.47 (total score range: 1–5), with scores for each section as follows: engagement, 4.00 (1–5); functionality, 4.50 (1–5); esthetics, 4.44 (1–5); and information, 4.95 (1–5).

## 4. Discussion

This study investigated the feasibility and effectiveness of utilizing CAT for individuals with MA use disorder. The results indicated slightly higher treatment retention in the experimental group than in the control group, but no significant clinical difference was observed, which may be attributed to factors such as the small sample size, treatment adherence, and some participants being referred for mandatory treatment. Additionally, participants' reluctance to use mobile phone messaging and unfamiliarity with integrating treatment into their daily lives may have resulted in dropouts. Our study found similar treatment retention rates compared with those of the Matrix Model, an intensive outpatient treatment program for patients with stimulant use disorders consisting of various treatment activities (e.g., an early recovery group, relapse prevention, and family involvement group therapy) (64, 66). Furthermore, the experimental group had significantly fewer MA-positive urine samples than the control group (19.5% vs. 29.6%, *F* = 9.116, *p* = 0.003). Similarly, in the original study investigating the Matrix Model, which followed 183 people who used MA, 19.3% of urine samples tested positive for MA after treatment (67). In another study that explored the therapeutic effects of a 12-week relapse prevention program in Taiwan, 69.0% of participants had an MA-positive urine screening result at least once throughout the program (64). Our findings also indicated a similar reduction in MA use compared with previous text-messaging interventions (48, 50, 68). The continuous format, immediate access to responses, and enhanced awareness concerning the importance of maintaining abstinence provided by CAT may have contributed to these better outcomes. Despite our data indicating that CAT may be less beneficial for individuals with more severe substance use disorders, polysubstance use, and low readiness to change, the high satisfaction scores indicate the feasibility and acceptability of CAT. At the end of treatment, most participants who remained in treatment agreed that CAT aided in their recovery and were satisfied with the program. Compared with the results of previous studies evaluating mHealth apps for the management of pain or diabetes, our CAT app was rated as having high quality, particularly regarding the quality of information, visual information, credibility, and evidence base (69, 70). Furthermore, the satisfaction survey results indicated favorable acceptance of the CAT. Overall, our study suggests that CAT offers benefits including accessibility, anonymity, personalization, cost-effectiveness, and adequate acceptance for patients with MA use disorder.

Despite the potential benefits of the use of chatbots in addiction treatment, several limitations of the study must be considered. First, CAT provided 24-h unlimited access to treatment materials and regular reminders, while the control group received limited contact. This may have resulted in a difference in the number of contacts received by the experimental and control groups over the 6-month period, which could have masked the therapeutic effects in both groups. Therefore, the mechanism of the therapeutic effects, such as the dose–response relationship and the influence of in-person or virtual contact should be further investigated. Second, the study sample was predominantly male, single, and employed, and the results may not be generalizable to populations with different demographic characteristics. The study population was limited to individuals with MA use disorder who were more accepting of technology-assisted treatment, motivated to participate in research, and willing to complete weekly assessments compared with the overall population of individuals with substance use disorder. The study participants may also have been underrepresented by individuals who are uncomfortable with using mobile phones or who use phones that run on the Android operating system, leading to unintentional selection bias. Therefore, our findings may not be generalizable to the entire population of patients with MA use disorder. By excluding individuals with acute exacerbation of mental illness, our sample may not be fully representative of the broader population of individuals struggling with methamphetamine use, who often have concurrent mental health issues or heavy alcohol consumption which could potentially impede their ability to participate fully in the study. This could potentially limit the generalizability of our findings. Third, the satisfaction survey was conducted only among the participants who completed the 6-month treatment and remained in the study until its end. Thus, the satisfaction of those who dropped out of the study before the end of treatment could not be evaluated. Furthermore, satisfaction with different treatments and components of the treatments were not evaluated. Moreover, the detection window for methamphetamine metabolites in urine typically ranges between 3 and 5 days post-consumption but can extend to a week for heavy or chronic users, contingent on factors such as the individual's metabolic rate, the drug's dosage, and the frequency of use. It is essential to note potential limitations tied to this monthly testing schedule. Specifically, sporadic or infrequent methamphetamine use may not be captured if usage does not coincide with the testing window. Furthermore, while immunoassay tests are highly dependable, they can occasionally yield false-positive results due to cross-reactivity with certain over-the-counter medications and other substances. Finally, although the present study was a longitudinal intervention study, the follow-up period of 6 months is relatively short compared with the DSM-5 criteria for full remission of 12 months.

Notably, this study recruited participants who were mandated addiction treatment. Despite the absence of significant differences in outcome variables among participants who were or were not referred for mandatory addiction treatment, our results revealed similar results among this population compared with those of other mandatory treatments for MA use disorder (71). However, the influence of mandatory treatment and its applicability for different populations require further investigation. Moreover, due to limitations in human resources, time, and participants' willingness to participate, a more in-depth assessment could not be conducted to gather additional data (e.g., childhood traumatic experiences and quality of therapeutic alliance). Only quantitative data assessing the feasibility and effectiveness of CAT were collected. Thus, future studies should qualitatively examine the details of users' experiences and the beneficial aspects of chatbots.

This study had several strengths, despite its limitations. We observed a significant reduction in MA use, according to urine test results, and identified potential factors related to the retention of treatment. Moreover, this is one of the few studies to investigate the use of chatbots for the treatment of individuals with MA use disorder, and it contributes to the growing body of knowledge related to mHealth. There is potential for a large-scale RCT in the future to further validate the effectiveness of CAT for methamphetamine use disorder. In an ideal scenario, the design of such a study should address several key factors. First, expanding the sample size would allow for greater statistical power and generalizability of the results. Including a diverse range of participants in terms of demographic characteristics (such as gender, age, and socioeconomic status), the severity of methamphetamine use disorder, and comorbid mental health conditions would also be beneficial to determine the effectiveness of the intervention in various subgroups. Second, the future RCT should consider a stratified randomization process to account for potential confounders such as the severity of substance use disorder, readiness to change, and other comorbidities. This would ensure balanced groups and increase the validity of the findings. Third, to comprehensively assess the efficacy of the CAT, a multifaceted evaluation approach should be adopted. Apart from urine tests, other measurements such as self-reported drug use, psychosocial functioning, quality of life, and participant satisfaction should be included. A longer follow-up period, ideally at 12 months, would also help to ascertain the long-term effects of the intervention. Furthermore, the issue of participant engagement with the chatbot should be addressed. Future trials could include strategies to promote regular interaction with the CAT, such as push notifications or rewards for consistent use. It would also be insightful to explore user experiences and preferences through qualitative methods to continually improve the chatbot design and functionality. Finally, given the potential for contamination between groups in digital interventions, it would be prudent to ensure that the control group receives a standardized level of care or alternative digital intervention to accurately determine the added benefit of the CAT. The design and implementation of chatbot-related interventions, including the type of therapy (individual or group) and the use of biosignals (such as heart rate variability, temperature, and blood pressure) to reflect an individual's craving status, could also be investigated. While no harmful or unintentional results of these interventions have been observed, studies regarding the privacy, ethics, and cost analysis associated with CAT are needed (72, 73). Moreover, the ability of chatbots to engage patients in the assessment of their own symptoms and daily functioning, increase patients' self-awareness and self-management of symptoms, improve patients' ability to identify triggers and track their own disease progression, and increase patients' willingness to seek care when necessary should also be investigated. Implementing these strategies in a future larger RCT could help further establish chatbot-assisted treatment as an effective, acceptable, and cost-efficient tool in managing methamphetamine use disorder. Overall, chatbots can provide individuals with access to care and support regardless of their location or schedule, without the stigma often associated with addiction treatment. Moreover, with the use of artificial intelligence algorithms, chatbots can tailor their conversations to an individual's specific needs and preferences and collect data on an individual's progress to provide insights and recommendations to healthcare providers. In the future, the possibility of using AI chatbots based on human nature for the treatment of patients with MA use disorders should be explored (74).

## 5. Conclusion

In this study, participants diagnosed with MA use disorder exhibited greater adherence to CAT, experienced favorable treatment outcomes, and reported favorable results in terms of treatment acceptance, indicating that CAT is feasible for individuals with MA use disorder. The use of chatbots holds promise as a method of providing immediate help and collecting valuable clinical information that can inform treatment decisions and monitor outcomes without imposing a significant burden on patients or providers. Moreover, the participants indicated that they were generally satisfied with receiving CAT.

The implementation of effective treatments for stimulant use disorder faces numerous barriers. For example, patients may experience a chaotic lifestyle, financial difficulties, a lack of social support, and stigmatization. These challenges hinder adherence to care, requiring innovative solutions. This study provides evidence of the effectiveness of using chatbots as a tool in the treatment of individuals with stimulant use disorder. However, a comprehensive understanding of the strengths and limitations of this technology in addiction treatment is warranted.

## Data availability statement

The raw data supporting the conclusions of this article will be made available by the authors, without undue reservation.

## Ethics statement

The studies involving human participants were reviewed and approved by IRB in Jianan Psychiatric Center (IRB-18-017). The patients/participants provided their written informed consent to participate in this study.

## Author contributions

LC-H: conceptualization, methodology, formal analysis, investigation, resources, data curation, writing-original draft, writing-review and editing, project administration, and funding acquisition. LG-H and YW-C: conceptualization, methodology, software, resources, and data curation. LY-H: formal analysis, investigation, and writing—review and editing.
